# Auditory contributions to postural and locomotor control: from basic research to clinical applications

**DOI:** 10.1007/s00221-026-07337-3

**Published:** 2026-06-22

**Authors:** Federica Cimmelli, Barbara La Scaleia, Isabelle Viaud-Delmon, Francesco Lacquaniti, Yury Ivanenko

**Affiliations:** 1 Laboratory of Neuromotor Physiology, Istituto di Ricovero e Cura a Carattere Scientifico Fondazione Santa Lucia, Rome, 00179 Italy; 2 Department of Systems Medicine, Center of Space Biomedicine, University of Rome Tor Vergata, Rome, 00133 Italy; 3 Department of Systems Medicine, University of Rome Tor Vergata, Rome, 00133 Italy; 4 CNRS, Ircam, Sorbonne Université, Ministère de la Culture, Sciences et Technologies de la Musique et du Son, STMS, Paris, 75004 France

**Keywords:** Auditory system, Vestibular system, Multisensory interactions, Rhythm generation, Human posture, Locomotion

## Abstract

Auditory information plays a critical role in human posture and locomotion. Beyond its obvious contribution to spatial awareness, sound provides continuous cues about body orientation, movement dynamics and environmental context. This review summarizes current knowledge on how auditory inputs modulate postural stability, locomotor kinematics and muscle coordination through multisensory integration with visual, somatosensory and vestibular systems. We highlight evidence from behavioural, neurophysiological and clinical studies showing that auditory features, for example coming from spatialised sounds, rhythmic stimulation and auditory feedback, can influence gait timing, trajectory control and balance recovery. A dedicated section explores the interaction between the auditory and vestibular systems, focusing on shared neural pathways in brainstem and cerebellum that contribute to motion perception and equilibrium. We also discuss alterations of auditory-motor coupling in neurological and sensory disorders, including vestibular deficits, Parkinson’s Disease and developmental coordination disorders, and we consider the implications for auditory-based rehabilitation. Understanding how sound informs and stabilizes human movement may open new perspectives for multisensory training, neuroprosthetic design and fall prevention strategies.

## Introduction

Human posture and locomotion depend on the continuous integration of multisensory information (Orlovsky et al. 1999; Peterka 2018; Ivanenko and Gurfinkel 2018; Mongeau et al. 2021; Lohse et al. 2022). Vision, somatosensation, and vestibular inputs are traditionally regarded as the principal sensory contributors to balance and gait control, yet growing evidence highlights that auditory information also plays a critical stabilizing and orienting role. Sound provides spatial, temporal, and contextual cues that help monitor body motion relative to the environment and anticipate perturbations. By encoding both self-generated and external acoustic signals, the auditory system contributes to sensorimotor prediction and adaptive movement regulation (Aytekin et al. 2008; Boyer et al. 2013; Bevilacqua et al. 2016).

These considerations fit within a broader framework in which movement control is understood as a multisensory process, relying on the coordinated contribution of peripheral receptors, brainstem circuits, and distributed cortical networks to maintain orientation, stability, and efficient locomotion in dynamic environments. Although the role of auditory information in these processes has historically received less attention than that of vision, somatosensation, and vestibular function, recent advances across behavioural neuroscience and mobile neurophysiology have begun to highlight how sound provides informative spatial and temporal structure relevant for movement control (King 2009; Morillon and Baillet 2017). Converging theoretical perspectives, including those synthesized in the sensory-motor theory of rhythm by Todd and Lee (2015), further suggest that auditory processing is closely intertwined with motor and vestibular systems, supporting the view that audition may contribute more broadly to sensorimotor regulation than traditionally assumed.

**Fig. 1:**
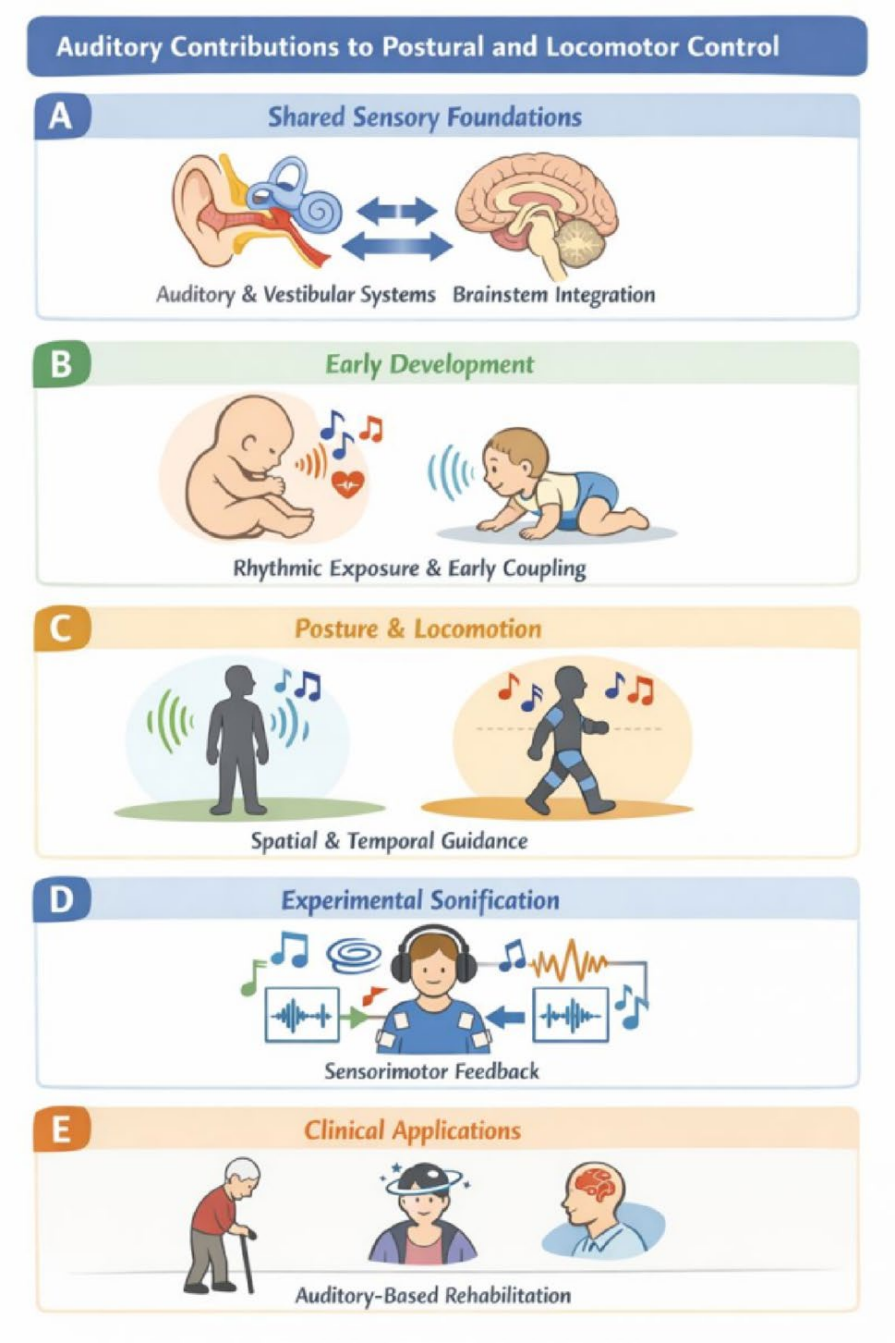
Multilevel contributions of auditory information to postural and locomotor control. Auditory cues interact with vestibular, visual, and somatosensory signals across multiple levels of organization (figure created with the assistance of an AI-based image generation tool and revised by the authors). **A** At peripheral and subcortical levels, auditory and vestibular systems share mechanosensory origins, overlapping pathways, and efferent control. **B** During development, prenatal and early postnatal exposure to rhythmic sound scaffolds auditory-motor and auditory-vestibular coupling. **C** In adulthood, auditory information acts as a spatial anchor for posture and a temporal organizer for locomotion. **D** Experimental sonification of self-movement allows controlled manipulation of action-effect loops involving auditory feedback and motor learning. **E** In neurological and sensory disorders, auditory cues provide external spatial or temporal scaffolds that support gait and balance rehabilitation

In this review, we examine the neurophysiological mechanisms and functional implications of auditory contributions to postural and locomotor control (schematically outlined in Fig. 1). We consider behavioural, neurophysiological, and clinical evidence illustrating how specific acoustic cues, such as those contained in environmental sounds, rhythmic stimulation, and auditory feedback, modulate balance, gait kinematics, and muscle coordination through multisensory integration with visual, somatosensory, and vestibular inputs. Special attention is given to the interaction between auditory and vestibular systems, as well as to alterations of auditory-motor coupling in neurological and sensory disorders. By synthesizing current findings from both basic and translational research, we aim to clarify how sound informs and stabilizes human movement and to outline perspectives for auditory-based interventions and rehabilitation strategies. This multisensory contribution of audition to movement control is rooted not only in higher-order cortical networks, but also in the shared peripheral and subcortical architecture of the auditory and vestibular systems, which we examine next.

## Auditory-vestibular interactions beyond anatomical proximity

At the mechanistic level, the auditory and vestibular systems share a common evolutionary origin in the labyrinth of the inner ear. Both derive embryologically from the otic placode, which gives rise to the membranous labyrinth, housing both the cochlea (hearing) and the semicircular canals and otolith organs (balance, postural tone, orientation). Early vertebrates had a single mechanosensory system specialized for detecting motion and vibration in the surrounding medium. The auditory system evolved later as a specialization of this primitive vestibular apparatus, tuned for airborne sound detection. In particular, both systems show frequency tuning: vestibular hair cells respond to low-frequency accelerations (< 5 Hz), corresponding to the natural dynamics of head and body motion (Benson et al. 1989), and these frequencies are extremely low relative to the auditory domain, where cochlear hair cells are tuned to acoustic vibrations in the ~ 20–20 000 Hz range (Moore 1996), representing a continuum of mechanical sensitivity across the whole inner ear labyrinth. Given this specialization, the vestibular system encodes head acceleration and orientation while the auditory system encodes spatial location and motion of sound sources via binaural (interaural time and level differences), monaural cues (due to the shading of the sound by the head, body, and pinnae), and the direct-to-reverberant energy ratio. Both systems are mechanosensory, relying on hair cells that transduce mechanical stimuli (vibration, acceleration, pressure) into neural signals via mechanoelectrical transduction.

Despite specialization, both systems show functional similarities and encode spatial and dynamic information, and often the impairment of one system affects the other. For instance, animal research has highlighted the interdependence of hearing and vestibular-related balance control. Consistent with this interdependence, animal studies show that auditory and vestibular functions share common susceptibilities: in mice, several deafness-related mutations produce combined cochlear and vestibular deficits (reviewed in Jones and Jones 2014), while in guinea pigs acoustic trauma induces parallel alterations in hearing thresholds and vestibulo-ocular reflexes (Fetoni et al. 2009). Human clinical studies corroborate these findings, demonstrating substantial cochlear-vestibular comorbidity across diverse aetiologies. Maudoux et al. (2022) reviewed evidence showing that deafness is frequently accompanied by vestibular deficits, reflecting the shared anatomy, vascularization, and developmental mechanisms of the cochlea and vestibular apparatus. In paediatric populations with genetic hearing loss, peripheral vestibular dysfunction occurs in approximately 52% of cases, affecting both syndromic (60%) and non-syndromic (46%) forms (Wang et al. 2021). Similarly, adults with sensorineural hearing loss exhibit vestibular abnormalities at variable but clinically significant rates: Pajor et al. (2002) reported abnormal electronystagmography in 72% of patients, while noise-induced hearing loss cohorts show vestibular dysfunction (Manabe et al. 1995; Wang and Young 2007; Kumar et al. 2010; Zuniga et al. 2012). Notably, age-related hearing loss may also be accompanied by vestibular weakness even in absence of overt predisposing factors for vestibulopathy (Kurtaran et al. 2016).

**Fig. 2:**
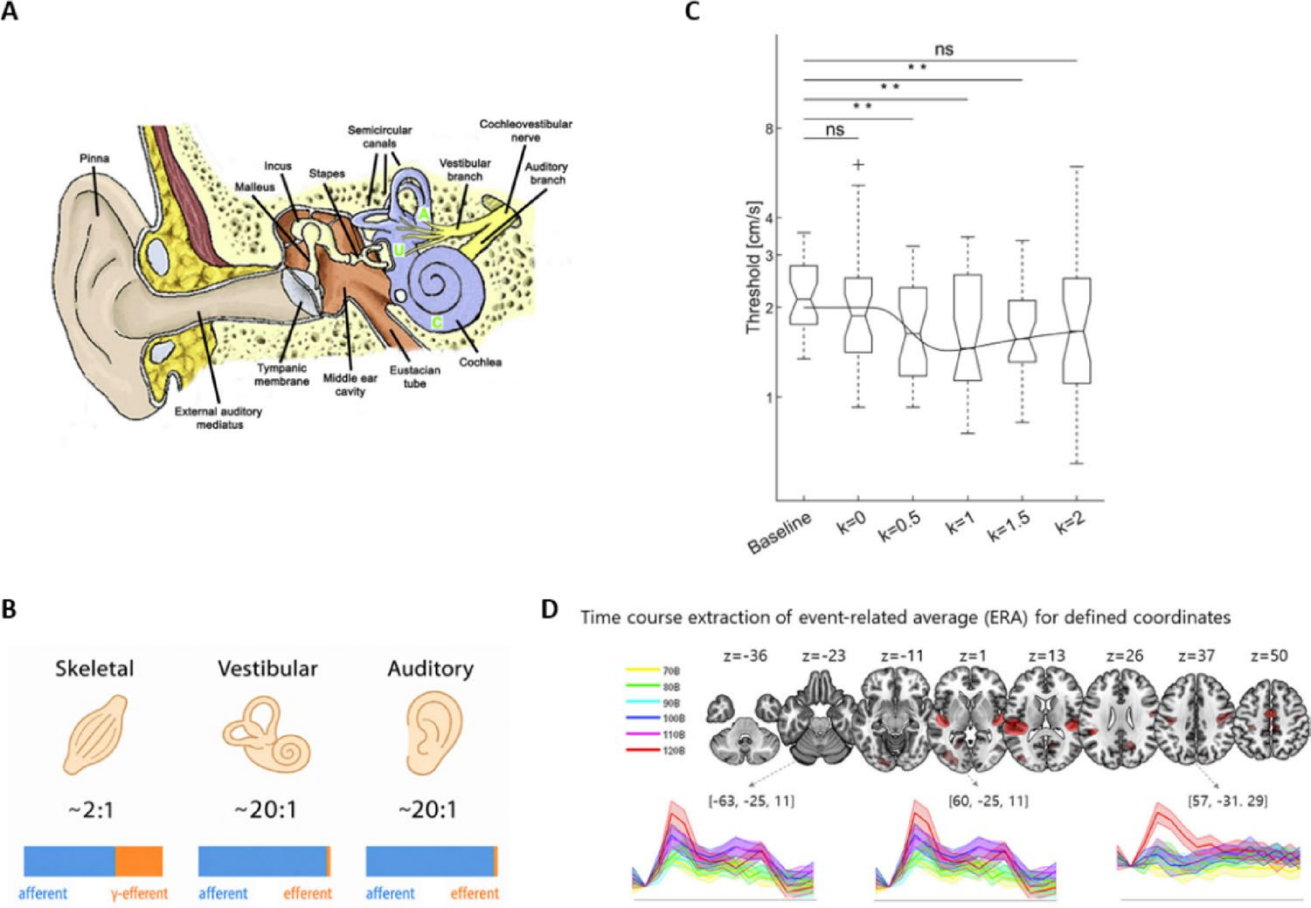
Multilevel auditory-vestibular interactions. **A** Anatomical organization of the human inner ear, illustrating the shared origin and close proximity of cochlear and vestibular end organs and their common routing through cranial nerve VIII (reviewed in Amar et al. 2022). **B** The human cochlear nerve contains ~ 30.000–50.000 fibres (Ota and Kimura 1980; Spoendlin and Schrott 1989; reviewed in Nadol 1988) with the vast majority afferent. Only a small proportion correspond to olivocochlear efferents arising from the superior olivary complex (reviewed in Guinan 2006, 2018). The vestibular nerve comprises ~ 15.000–23.000 fibres (Rosenhall 1972) of which ~ 2–5% are efferent-similar in proportion to cochlear efferents but fewer in absolute number. For comparison, mixed peripheral nerves innervating skeletal muscle contain sensory afferents together with α-, β- and γ-motor efferents. Unlike α-motoneurons, which directly drive force production, γ-motor fibres regulate muscle spindle sensitivity and are therefore functionally analogous to vestibular and auditory efferent systems. Bars schematically illustrate the approximate relative proportions of sensory afferents and modulatory efferents across systems. Whereas auditory and vestibular nerves contain only sparse efferent innervation (schematically depicted as ~ 20:1 afferent/efferent ratio), proprioceptive systems associated with skeletal muscle exhibit comparatively denser γ-motoneuron innervation relative to sensory afferents (~ 2:1 afferent-to-γ ratio, varying with muscle type; Boyd and Davey 1968; Matthews 1974; Prochazka 1996). **C** Whole‑body motion discrimination thresholds (La Scaleia et al. 2023), showing how low‑amplitude stochastic mechanical perturbations (k = 0.5–1.5) enhance vestibular sensitivity relative to baseline. Boxplots display median, interquartile range, and 5th–95th percentiles; the overlaid curve illustrates the characteristic inverted‑U profile of stochastic resonance. **D** Functional MRI maps (Oh et al. 2018), showing the cortical locations used for time‑course extraction. Axial slices display three representative coordinates belonging to the auditory, multisensory auditory‑vestibular, and vestibular networks. For each coordinate, the corresponding plot shows the average BOLD time course across six sound‑pressure levels (70–120 dB), illustrating how response amplitude varies with stimulus intensity in each network

In humans, the inner ear constitutes a highly specialized sensory organ embedded within the temporal bone (Fig. 2A), comprising two anatomically contiguous subsystems: the cochlea and the vestibular apparatus. Despite their traditional conceptual separation, both structures share the same endolymphatic milieu and are organized within the continuous osseous and membranous labyrinth. Hair cells in both cochlear and vestibular organs use similar molecular machinery: mechanotransduction channels, synaptic ribbon structures, glutamatergic neurotransmission, and afferent connections via the VIII cranial nerve (vestibulocochlear nerve) (Longridge 2023). The vestibular nuclei and cochlear nuclei in the brainstem are closely interconnected and project to shared targets, such as the reticular formation, cerebellum, and thalamus, supporting multisensory integration for balance and spatial orientation. Anatomical and physiological studies further show that vestibular inputs reach both the ventral and dorsal cochlear nuclei, and that neurons in the lateral and medial vestibular nuclei send glutamatergic projections to the dorsal cochlear nucleus (Smith 2012). These pathways position the dorsal cochlear nucleus as a multisensory hub that integrates auditory, somatosensory, and vestibular signals, supporting functions such as sound localization during head movements and contributing to plastic changes following auditory damage. The inferior colliculus and cerebellum receive convergent auditory and vestibular inputs, enabling cross-modal calibration between sound localization and self-motion cues.

Beyond their afferent organization, both the vestibular and auditory systems also exhibit efferent modulation, allowing central control of peripheral sensory processing. Efferent control is traditionally more investigated for skeletal muscles, where the percentage of efferent fibres is high (Fig. 2B). This organization suggests that proprioceptive systems rely more heavily on peripheral efferent calibration than auditory and vestibular systems, possibly reflecting the continuously changing mechanical properties of muscles during movement. Nevertheless, despite being few, both the vestibular and auditory systems also possess descending efferent pathways that modulate peripheral sensory processing in a context-dependent manner. In the vestibular apparatus, efferent neurons, originating from the brainstem, project bilaterally to hair cells and primary afferents in the semicircular canals and otolith organs. These cholinergic efferents adjust receptor sensitivity and afferent firing, contributing to adaptive gain control, suppression of self-motion artefacts, and stabilization of perception during active behaviours such as locomotion or gaze shifts (reviewed in Holt et al. 2011; Highstein 1991; Mathews et al. 2017). Recent comparative work also indicates that vestibular efferent influence became relatively reduced in mammals, where it predominantly targets irregular afferents, compared with the stronger and more widespread efferent control characteristic of non-mammalian vertebrates (Cullen and Wei 2021). A parallel mechanism operates in the cochlea through the olivocochlear efferent system, comprising medial fibres that innervate outer hair cells and lateral fibres that target auditory nerve dendrites. This system, also predominantly cholinergic, modulates cochlear amplifier gain, enhances signal detection in noisy environments, provides protection against acoustic overstimulation, and contributes to perceptual and protective functions in a state-dependent manner (reviewed in Guinan 2018; Lauer et al. 2022). Thus, both systems employ real-time efferent feedback to align sensory input with behavioural state and cognitive demands. While cochlear efferent function is well documented in humans (reviewed in Guinan 2006), evidence for vestibular efferent modulation comes largely from animal physiology, where efferent activation alters afferent firing properties but its functional implications at the behavioural level remain to be established (Goldberg and Fernandez 1980).

Finally, the close anatomical continuity of the auditory and vestibular end organs, both relying on similar mechanoelectrical transduction mechanisms, creates natural pathways for direct interaction between the two systems. In some cases, acoustic stimulation can directly activate vestibular afferents. Clinically, this is demonstrated by the fact that high-intensity sounds evoke vestibular responses detectable via vestibular evoked myogenic potentials (VEMPs): cervical VEMPs (cVEMPs) to assess saccular function through the vestibulo-collic reflex, and ocular VEMPs (oVEMPs) to evaluate the utricular function and the superior vestibular nerve pathway (reviewed in Colebatch et al. 2016; Curthoys et al. 2017, 2025; Rosengren and Colebatch 2018). In addition, fMRI studies reveal that intense acoustic stimulation elicits robust BOLD responses in vestibular and multisensory cortical regions (Oh et al. 2018) (Fig. 2D). In this case, participants listened to sounds presented at six increasing intensities (70–120 dB), covering levels that are purely auditory as well as intensities known to activate the sacculus. The authors then examined how the BOLD signal changed in auditory, multisensory, and vestibular cortical regions by extracting the average time course of the response at each intensity. Linear increases across sub-threshold levels reflected classical auditory processing, whereas a disproportionate enhancement at 120 dB indicated recruitment of vestibular pathways, consistent with sacculo-mediated auditory-vestibular activation. Overall, the results show an intensity-dependent recruitment of vestibular pathways: when acoustic energy exceeds the mechanical threshold of otolithic receptors, then a vestibular contribution emerges. Beyond clinical diagnostics, recent animal work has demonstrated that specific stimulus parameters, such as rise time, frequency content, and acceleration profile, differentially modulate vestibular output, as shown by simultaneous measurements of macular mechanics and vestibular compound action potentials in guinea pigs (Pastras et al. 2023). Pathological conditions can exaggerate this auditory-vestibular coupling. “Third-window” syndromes, such as superior semicircular canal dehiscence, create abnormal pathways for sound and pressure transmission within the labyrinth, allowing acoustic energy to directly excite vestibular end organs. Patients with these conditions exhibit sound- or pressure-induced vertigo and characteristic eye movements (Minor et al. 1998; Mong et al. 1999), a clinical manifestation of the same mechanoelectrical sensitivity that underlies normal auditory-vestibular interactions. Radiologic and biomechanical studies have clarified that these syndromes arise from an additional compliant opening in the otic capsule, which alters fluid impedance and produces pathological pressure gradients across the semicircular canals (reviewed in Ho et al. 2017; Iversen and Rabbitt 2020). Related phenomena, including the classical Tullio effect and Hennebert sign, further illustrate how acoustic or pressure energy can directly drive vestibular activation by inducing endolymph flow and cupular deflection within the affected canal, when labyrinthine mechanics are disrupted (Wade et al. 2023).

Evidence from electrophysiology further shows that vestibular activation contributes to cortical auditory responses. When acoustic stimulation exceeds vestibular thresholds, cortical auditory evoked potentials acquire additional mid-latency components, such as the N42/P52 complex, reflecting vestibular-driven activity in temporo-parietal and cingulate regions (Todd et al. 2014). Intracranial recordings also demonstrate that the posterior insula responds to auditory stimulation and likely participates in auditory-vestibular convergence (Citherlet et al. 2020). These findings indicate that vestibular inputs reach cortical auditory pathways and shape early sensory processing beyond the brainstem level.

In addition to high‑intensity sound effects on the vestibular system (reviewed in Curthoys et al. 2017), a substantial body of work has examined how auditory and vestibular signals interact to shape spatial perception. Vestibular stimulation can bias auditory localization (Lewald and Karnath 2000), eye position determines auditory-vestibular integration during whole‑body rotation (Van Barneveld and John Van Opstal 2010), and vestibular cues are continuously combined with proprioceptive and haptic information to maintain spatial orientation (Lackner and DiZio 2005). More recent work has shown that these interactions are essential for maintaining a stable representation of auditory space during self-motion. When the head rotates, accurate spatial updating of sound location relies predominantly on vestibular signals, complemented by proprioceptive and efference-copy cues (Genzel et al. 2016). Moreover, auditory and vestibular cues are combined in a frequency-dependent manner during self-motion perception: low-frequency acoustic information enhances estimates of body motion, whereas higher-frequency components are dominated by vestibular input (Shayman et al. 2020). By contrast, a less explored field concerns how low‑intensity (auditory or vestibular) stimulation modulates vestibular thresholds. Recent experimental work shows that vestibular processing is shaped by the physical characteristics of incoming signals, irrespective of whether they originate from mechanical or acoustic sources. La Scaleia et al. (2023) in their experiment quantified vestibular motion discrimination thresholds by delivering whole-body perturbations along the anteroposterior axis. Crucially, the perturbations were embedded in stochastic mechanical noise of six amplitudes (baseline, k = 0, 0.5, 1, 1.5, 2), allowing the authors to estimate the minimum acceleration required for reliable direction discrimination under each noise condition. They found a characteristic reduction in thresholds exclusively at intermediate noise levels (k = 0.5–1.5), whereas higher noise levels (k = 2.0) yielded no perceptual benefit, indicating that otolithic processing benefits from the addition of low-amplitude mechanical fluctuations. This non-monotonic, U-shaped relationship between noise intensity and perceptual improvement is consistent with stochastic resonance mechanisms. Stochastic resonance (SR) refers to a phenomenon in nonlinear systems whereby small-amplitude random noise enhances the detection and transmission of weak signals (Benzi et al. 1982; Gammaitoni et al. 1998). In this framework, low-amplitude noise improves the detectability of subthreshold vestibular signals by increasing the probability that the combined signal-noise input crosses the perceptual threshold (Fig. 2C). Given the functional importance of adjusting vestibular thresholds for movement perception and control, it would be interesting to study the effects of auditory stimulations on the ‘tonic’ state of the vestibular system, although this issue remains to be thoroughly investigated.

In summary, the auditory and vestibular systems function as closely related mechanosensory “sister systems”, sharing evolutionary origins, anatomical architecture, and core physiological principles. Both develop early, rely on hair-cell transduction, and project to partially overlapping central circuits that integrate sensory and motor information for orientation, balance, and movement. Their functional permeability extends well beyond anatomical proximity. This convergence helps explain the frequent co-occurrence of auditory and vestibular deficits and points to shared developmental mechanisms. Together, these systems support postural control, spatial orientation, and navigation, often compensating for one another when one modality is degraded. These shared mechanosensory principles and central pathways are already operative early in life, providing the biological substrate upon which auditory-motor coupling emerges during prenatal and postnatal development.

## Auditory-motor coupling in early development

From a developmental perspective, the auditory and vestibular systems originate from the same otic placode, differentiating into cochlear and vestibular structures that share vascularization and innervation (Johnson Chacko et al. 2019). Although the vestibular apparatus reaches functional maturity earlier, supporting orientation and reflexes at birth, the auditory system becomes responsive in the late second trimester, when fetuses begin to perceive intrauterine sounds (reviewed in Graven and Browne 2008; Yetkin et al. 2023). Sound travels through the amniotic fluid and stimulates the fetal auditory system via the bone conduction pathway rather than through the external and middle ear systems (reviewed in Gerhardt and Abrams 1996). Comparative studies have further highlighted the evolutionary conservation of vestibular development across vertebrates, underscoring its precocity relative to auditory maturation (reviewed in Mackowetzky et al. 2021).

Given that rhythmogenesis is the core of locomotor pattern generation circuits (Orlovsky et al. 1999), it is interesting to note how fetuses and newborns are exposed to a rhythmic acoustic environment, which may in turn shape the developing nervous system’s capacity to perceive and produce rhythmic movement. During fetal life, auditory experience occurs largely through bone and tissue conduction because the external and middle ear are not yet air-filled, with external sounds reaching the uterus in a filtered and attenuated form due to propagation through maternal tissues (Gélat et al. 2025). Prenatal exposure to rhythmic acoustic patterns, such as maternal heartbeat, speech prosody, and musical stimuli, has been shown to shape neural circuits involved in timing and motor coordination, with effects observable in neonatal behaviour (Movalled et al. 2023). Consistent with this prenatal rhythmic exposure, auditory-motor coupling begins to emerge before birth, although slightly later than tactile and vestibular motor coupling (reviewed in Craighero 2024). After birth, the transition to a richer and more directly transmitted acoustic environment further strengthens this link: newborns already display sensitivity to rhythmic structure and can generate organized motor rhythms (reviewed in Provasi et al. 2014). Even in the perinatal period, infants modulate their spontaneous motor tempo in response to rhythmic auditory stimulation, revealing an early connection between rhythm perception and motor timing. These early couplings provide a developmental substrate for later synchronization abilities, including coordinated stepping and gait entrainment to external sounds, and illustrate how auditory cues begin to influence posture and locomotion from the earliest stages of life (reviewed in Provasi et al. 2014).

**Fig. 3:**
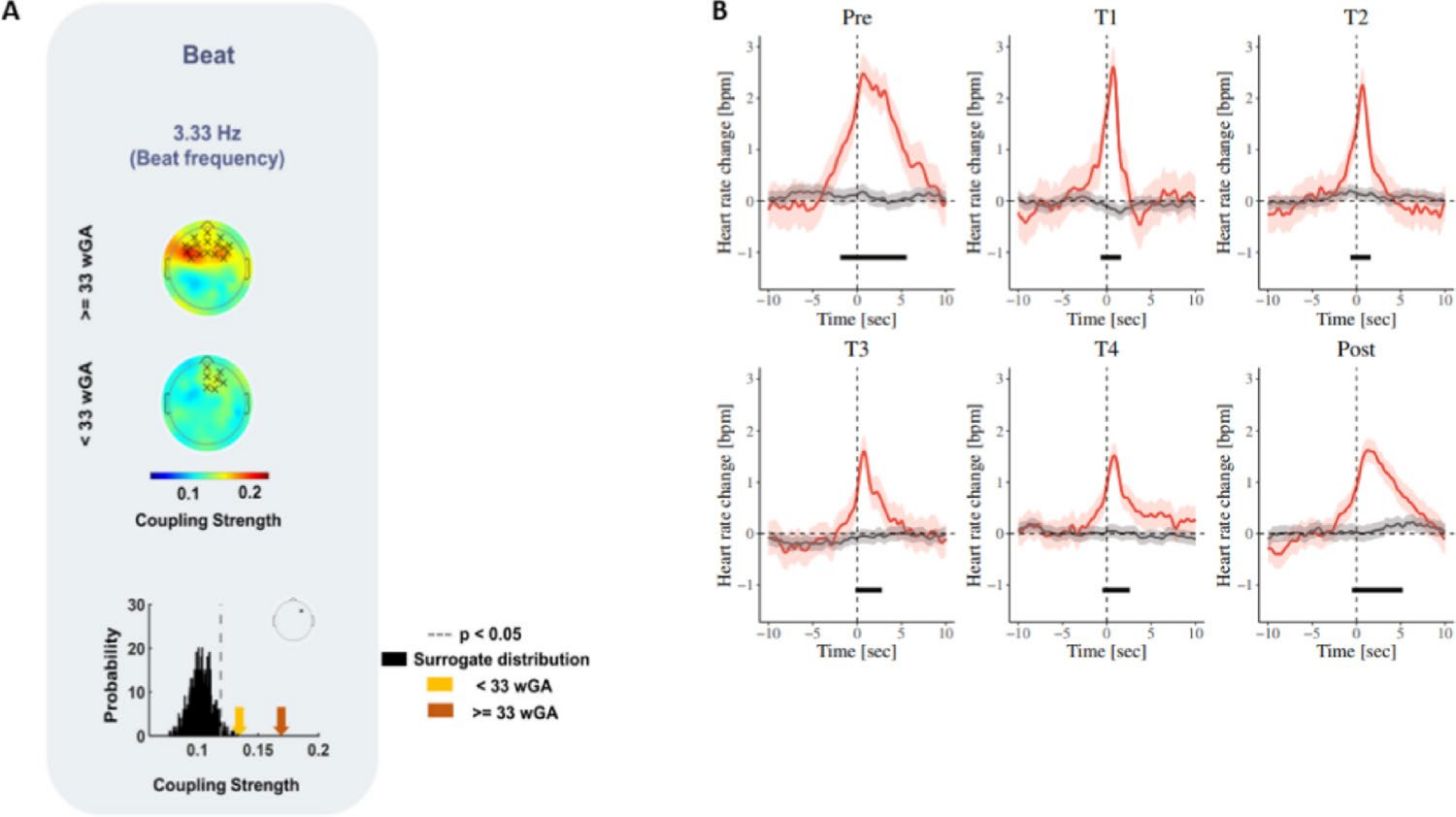
Early auditory-motor and auditory-autonomic coupling in fetal and infant development. **A** EEG topographical maps (Saadatmehr et al. 2025) showing fetal neural synchronization to rhythmic sound. Coupling strength between brain activity and beat (3.33 Hz) or meter periodicities (duple: 1.67 Hz; triple: 1.11 Hz) is displayed separately for younger (< 33 weeks gestational age) and more mature fetuses (≥ 33 weeks). Black clusters indicate electrodes significantly synchronized with the corresponding rhythmic frequency. **B** Event‑related heart‑rate responses (Shinya et al. 2022), aligned to spontaneous limb movements across the Pre phase, four Test sub‑phases (T1-T4), and Post phase. The Pre and Post phases correspond to baseline periods without auditory feedback, whereas T1-T4 represent successive segments of the Test phase during which infants experienced movement‑contingent sound feedback. Red traces show the average heart‑rate change time‑locked to movement onset, while grey traces represent surrogate waveforms obtained by time‑shuffling movement onsets. Shaded areas denote 95% confidence intervals, and black horizontal bars mark time windows with significant differences between original and surrogate data (adjusted *p* < 0.05)

Neurophysiological and behavioural data indicate that rhythm sensitivity emerges before full cortical maturation. Premature neonates show differentiated brain responses to beat and meter, revealing selective sensitivity to regular pulse and metric structure, which implies an early predisposition of auditory-motor systems to align with temporal regularities (Edalati et al. 2023). Complementarily, fetal recordings in the last trimester demonstrate tracking from basic beat to nested hierarchical temporal structures (Saadatmehr et al. 2025), supporting the notion that rhythmic encoding precedes birth and provides a scaffold for sensorimotor coupling (Fig. 3A). In the perinatal window, infants modulate spontaneous motor tempo in response to rhythmic auditory stimulation, consistent with early links between rhythm perception and motor timing and with the involvement of auditory cortex, basal ganglia, and cerebellum in temporal coding (reviewed in Provasi et al. 2014).

Beyond overt motor adjustments, recent work shows that the emergence of auditory-motor coupling in early infancy recruits not only cortical and behavioural mechanisms but also anticipatory autonomic regulation. Using a contingent auditory‑feedback paradigm in 3‑month‑old infants, Shinya et al. (2022) demonstrated that spontaneous limb movements become more frequent and temporally regular when each movement triggers a sound, indicating the formation of an action-effect association. Crucially, cardiovascular recordings revealed that the heart‑rate increase that normally precedes spontaneous movements is progressively suppressed and temporally realigned as infants learn the auditory contingency. This shift in event‑related heart‑rate dynamics reflects an emerging predictive control loop in which the infant’s autonomic system prepares for the expected sensory consequence of self‑generated action (Fig. 3B). Such anticipatory regulation suggests that auditory-motor coupling is scaffolded by multisystem integration from the earliest stages of development, well before the maturation of voluntary motor control.

Mechanistically, converging evidence maps how auditory timing signals interact with motor networks to enable entrainment and synchronization. Auditory cortex extracts temporal regularities; basal ganglia contribute to beat-based timing and sequence structure; cerebellum supports prediction and error correction; and motor cortex implements the aligned movement, together forming a distributed circuit that translates rhythmic sound into coordinated action (reviewed in Pranjić et al. 2024). Complementary neuroimaging work in adults shows that producing complex rhythmic structures, such as polyrhythms, recruits an extended network including supplementary motor area (SMA), inferior parietal regions, cerebellum, and basal ganglia, highlighting the integrative transformations required to map auditory rhythms onto motor output (Thaut et al. 2008). Behaviourally, infants produce systematic rhythmic motor responses while listening to rhythmic speech, exemplifying real-time sensory-motor integration and highlighting that language prosody can act as a natural entrainment driver early in life (Boll-Avetisyan et al. 2024).

Finally, dynamical systems modelling suggests that early rhythm perception is not purely auditory: vestibular inputs – arising from self-motion and passive body oscillations – interact with auditory signals to stabilize and amplify beat extraction without requiring mature cortical control (Tichko et al. 2021). This multisensory account aligns with the developmental trajectory outlined in the introduction: an early-functional vestibular apparatus and progressively responsive auditory system together support rapid emergence of auditory-motor synchronization, laying groundwork for later gait entrainment and postural modulation by sound.

## The auditory system as a spatial anchor in postural control

At the functional level, the early emergence of auditory-motor and auditory-vestibular coupling has clear consequences for adult postural control, where sound can act as an external spatial reference to stabilize upright stance. Postural stability emerges from the dynamic integration of vestibular, visual, and somatosensory inputs, each with a distinct contribution: the vestibular system provides an internal gravitational reference, vision provides an external spatial map, and proprioception provides body-state information (Eysel-Gosepath et al. 2016; Chaudhary et al. 2022; Appiah-Kubi et al. 2024). Numerous studies have shown that the central nervous system (CNS) continuously recalibrates the relative weight of these inputs (“sensory reweighting”), ensuring adaptation even in conditions of conflict or deficit (Peterka 2018; Lohse et al. 2022).

While the contributions of vision, somatosensation, and vestibular inputs to balance are well established, the role of hearing has historically been underestimated. As highlighted in the review by Lubetzky et al. (2020), a growing body of experimental evidence shows that sound can operate as a spatial anchor, particularly when other sensory channels are degraded. In healthy adults, static broadband noise delivered through external speakers reliably reduces postural sway under challenging conditions such as eyes‑closed stance, narrow bases of support, or foam surfaces (Zhong and Yost 2013). Not all sounds exert the same influence. The effect of sound on postural stability depends strongly on the physical characteristics of the acoustic signal. Park et al. (2011) showed that pure tones at higher frequencies (3–4 kHz) can increase antero‑posterior sway, indicating that certain frequency bands may interfere with postural control. The authors suggested that this destabilizing effect may reflect interactions between auditory and vestibular processing, possibly combined with increased attentional or arousal responses elicited by high-frequency sounds. In contrast, Siedlecka et al. (2015) reported that other high‑frequency or spectrally richer sounds can reduce sway area, suggesting that auditory effects on balance are not uniformly destabilizing but vary with the spectral content of the stimulus.

Beyond these basic acoustic properties, the spatial organisation of auditory cues plays a critical role. Rich auditory scenes further enhance stability, suggesting that spatially informative sound fields can be exploited by the postural control system (Gandemer et al. 2017). Consistent with this, Stevens et al. (2017) demonstrated that distributing sound sources around the listener improves postural control, particularly in individuals with greater baseline imbalance standing in the dark. Similar benefits are observed in individuals with vestibular dysfunction, who show increased sway when auditory cues are removed and marked improvements when stationary, world-fixed sounds are available (Vitkovic et al. 2016; Maheu et al. 2019). More recent work shows that the spatial position of a sound source contributes to balance: easily localizable frontal or rear sounds provide more reliable anchoring than laterally positioned stimuli, especially when visual or somatosensory cues are degraded (Otsuka et al. 2025; Paromov et al. 2025).

However, when auditory cues are head‑referenced rather than world‑fixed, they no longer provide spatial anchoring, yet they may still influence postural control through mechanisms unrelated to spatial localization. For instance, Ross and Balasubramaniam (2015) observed postural stabilization effects by presenting continuous white noise through headphones. Their work demonstrated that this stationary noise reduces postural fluctuations even without visual input, suggesting that sound can support balance independently of vision despite the minimization of externally localized spatial cues. Extending this approach, Tokiwa et al. (2024) showed that calibrated white noise continues to attenuate sway even when both vision and lower‑limb somatosensory feedback are degraded, highlighting the robustness of auditory contributions under multisensory challenge. These findings align with the systematic review by Minino et al. (2023), which showed that external sensory stimulations, including acoustic cues, modulate postural control in healthy adults, and with meta‑analytic evidence indicating that auditory cues exert a small‑to‑moderate but significant stabilizing effect across diverse populations and tasks (reviewed in Zarei et al. 2022).

Additional evidence from dual‑task paradigms in individuals with intellectual disabilities indicates that auditory environments shape the trade‑off between postural stability and cognitive performance, with some soundscapes facilitating balance under cognitive load and others acting as additional sensory stressors (Jouira et al. 2025). Importantly, auditory processing rarely acts in isolation. Combined auditory and visual stimulation produces greater stabilization than either modality alone (Hojan-Jezierska et al. 2022), and somatosensory signals modulate auditory processing at multiple levels, reflecting a flexible sensory hierarchy in balance regulation (reviewed in Lohse et al. 2022).

Collectively, these findings confirm that the auditory system is not a passive sensory channel but an active stabilizing force within the multisensory network. This conclusion is further supported by clinical evidence showing that restoring auditory input improves balance: in older adults with hearing loss, wearing hearing aids reduces postural sway compared to unaided conditions (Negahban et al. 2017; Ninomiya et al. 2021), and removing hearing aids leads to measurable increases in sway (Kolasa et al. 2025). These results indicate that access to reliable auditory information contributes to postural stability even in everyday listening environments. This dynamic interplay provides the foundation for examining how auditory and vestibular systems interact within shared anatomical and functional substrates. If audition can anchor the body during quiet stance, its contribution becomes even more critical during locomotion, where postural control must be continuously updated in time with rhythmic movement.

## Auditory-motor coupling in locomotion

Across evolution, the coupling between auditory processing and locomotor control has emerged as a fundamental organizational principle of vertebrate sensorimotor systems. Although locomotion is often conceptualized as the output of spinal and brainstem central pattern generators (Orlovsky et al. 1999; D’Elia and Dasen 2018), comparative neurobiology shows that these circuits have always been embedded within a broader multisensory architecture capable of integrating acoustic information to refine timing, orientation, and adaptive movement (Mongeau et al. 2021). Early bilaterians already possessed genetically specified motor circuits organized around rhythmic locomotor patterns and strongly shaped by sensory and neuromodulatory inputs (Bennett 2021). In aquatic vertebrates, sound and vibration cues modulate swimming patterns, escape responses, and orienting behaviours, demonstrating that auditory-motor interactions long predate terrestrial gait. Teleost fishes, amphibians, and birds exhibit robust acoustically driven motor responses, from phonotaxis to startle reflexes and postural adjustments, highlighting the evolutionary continuity of auditory influences on movement (reviewed in Ladich and Winkler 2017). These ancestral mechanisms illustrate that sound has always served as a temporally precise and spatially informative signal capable of shaping motor output.

**Fig. 4:**
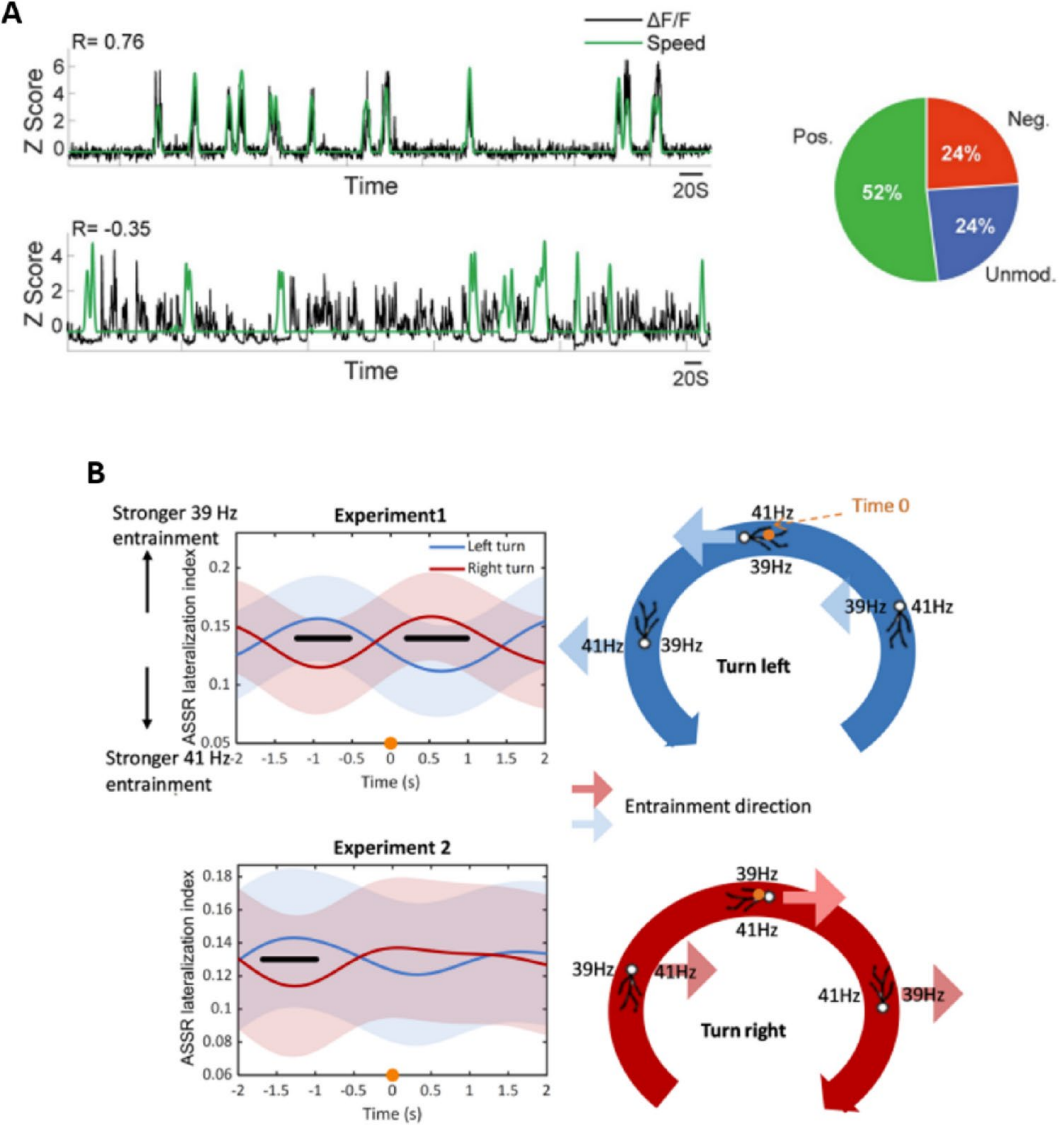
Auditory-motor coupling during locomotion in rodents** A** and humans** B**. **A** Neural encoding of locomotion speed in mouse auditory cortex (Vivaldo et al. 2023). On the top: example of auditory cortical neuron showing a strong positive correlation between its normalized calcium‑fluorescence signal (ΔF/F) and the animal’s running speed during an imaging session. On the bottom: example neuron exhibiting a negative correlation with locomotion speed. Right panel illustrates proportions of L2/3 neurons with significant positive, significant negative, or non‑significant correlations with locomotion speed, highlighting the diversity of locomotion‑related modulation across the population. **B** Locomotion‑dependent modulation of auditory steady‑state responses (ASSRs) in humans during natural walking (Chen et al. 2025). Participants walked along an 8‑shaped path while continuous dichotic amplitude‑modulated tones were presented (39 Hz to the left ear, 41 Hz to the right ear). Mobile EEG was recorded throughout. Turning events were extracted from ankle‑mounted gyroscope data, and ASSR power was time‑locked to the midpoint of each turn (0 s). The lateralization index reflects the relative strength of left‑ versus right‑ear entrainment. Left panels show time‑resolved lateralization index of the ASSR aligned to the midpoint of left (blue) and right (red) turns in two experiments. Positive values indicate stronger 39‑Hz entrainment (left ear), negative values stronger 41‑Hz entrainment (right ear). Black bars mark time windows with significant differences between trajectories (FDR‑corrected). Right panels display schematic walking path showing the spatial arrangement of the 39‑Hz and 41‑Hz ear‑specific modulations relative to the direction of movement. These experiments highlight shifting lateralized auditory attention according to the direction of movement

In mammals, this evolutionary logic is preserved and expanded. Rather than being suppressed during movement, the auditory system dynamically integrates locomotor signals to maintain perceptual stability and guide action. Recent work in rodents demonstrates that locomotion profoundly reorganizes auditory cortical activity: instead of a uniform reduction in sound-evoked responses, neuronal ensembles encode both acoustic features and the animal’s locomotion speed, forming a joint “sound-in-motion” representation (Vivaldo et al. 2022, 2023). This reorganization does not reflect a loss of sensory information but the emergence of an additional motor‑related signal within auditory cortex. During locomotion, ongoing activity increases and reliably tracks the animal’s running speed, providing a continuous representation of locomotor state that coexists with sound‑evoked responses (Fig. 4A). Rather than degrading auditory processing, this elevated baseline compresses response amplitudes while preserving the discriminability of acoustic events at the ensemble level. As a result, auditory cortical populations form a joint code for sound and locomotion speed, specifically tracking the animal’s continuous running velocity, supporting stable sound perception during movement and revealing the auditory cortex as an active sensorimotor integrator rather than a passive sensory relay. In the context of auditory-motor coupling, a useful distinction concerns whether rhythmic synchronization emerges spontaneously or requires explicit training. Several non‑human mammals can learn to align their movements to a beat, but only after extensive reinforcement. Macaques, for example, can acquire predictive beat‑based synchronization when temporal prediction, beat identification, and sensorimotor mapping are strengthened through training (Rajendran et al. 2025), and even robust auditory-motor associations in motor cortex support sequence learning rather than spontaneous beat identification (Archakov et al. 2020). Similarly, a California sea lion can be conditioned to synchronize head movements to metronomic and musical rhythms (Cook et al. 2013), whereas a harbor seal pup adopts a different strategy, producing antisynchronous calls rather than entraining to the beat (Ravignani 2019). In contrast, some vocal‑learning species exhibit spontaneous synchronization without explicit training. The sulphur‑crested cockatoo Snowball aligns his movements to music across multiple tempi in a motivation‑driven manner (Patel et al. 2009), illustrating that specialized auditory‑motor pathways in vocal learners can support effortless beat tracking.

Human neurophysiology further supports the existence of an intrinsic auditory-motor architecture. Resting‑state MEG data show that auditory and motor cortices are not only phase‑coupled across theta, alpha, and beta bands, but also exchange information bidirectionally: phase‑transfer entropy reveals preferential motor‑to‑auditory flow in theta and beta rhythms, and auditory‑to‑motor flow in alpha and gamma bands (Bedford et al. 2025). This frequency‑specific reciprocity indicates that auditory and motor regions form a coherent, dynamically organized network even in the absence of overt behaviour.

Importantly, during active movement this intrinsic architecture becomes functionally expressed: when humans walk, auditory cortical entrainment is not merely preserved but dynamically reweighted according to the direction of locomotion. Mobile EEG recordings show that walking alters auditory cortical dynamics, enhancing entrainment to rhythmic sounds and shifting lateralized auditory attention according to the direction of movement (Chen et al. 2025). The lateralization of the auditory steady‑state response shifts systematically with the turning trajectory, revealing a movement‑dependent modulation of spatial auditory gain (Fig. 4B). Such intrinsic and behaviourally expressed bidirectional coupling provides a mechanistic scaffold for rapid auditory-motor interactions during active behaviour, supporting temporal prediction, rhythmic entrainment, and the coordination of movement with auditory cues, optimizing environmental monitoring and navigation. Thus, neurophysiological studies indicate that locomotion itself modulates auditory processing, suggesting a bidirectional relationship. Another study using EEG frequency tagging demonstrated that rhythmic auditory and audiovisual inputs significantly enhanced neural entrainment in the sensorimotor cortex compared to visual-only inputs, highlighting the specialized role of sound in stabilizing and organizing the neural processing of walking-related movements (Matamala-Gomez et al. 2026). Beyond these functions, experimental work shows that the acoustic consequences of movement can themselves reshape motor behaviour: coupling one’s actions to self-generated musical feedback reduces perceived exertion and reorganizes motor patterns during strenuous whole-body exercise (Fritz et al. 2013). How we move might also influence how we hear (Phillips-Silver and Trainor 2005, 2008). Together, these findings support a multisensory and reciprocal framework: auditory cues guide movement, while movement‑generated sounds are reintegrated into action control, closing an auditory-motor loop (Boyer et al. 2013).

These findings suggest that auditory-motor coupling is not an emergent property of higher cognition but a deeply conserved feature of vertebrate neurobiology, shaped by the ecological demands of navigating dynamic environments.

## Sonification of self-movements as a tool to study multi-model interactions and motor learning

The intrinsic coupling between auditory processing and locomotor control provides a powerful opportunity for experimental manipulation. Sonification of self-movement allows these multisensory action-effect loops to be isolated, amplified, and systematically studied.

Sonification of limb and body movements is becoming a useful tool for investigating action-effect associations in which self-generated movements produce systematic auditory consequences, and has been shown to change behaviour and enhance perceptual accuracy (Effenberg 2004). Foundational work by Reh and colleagues (Reh et al. 2019; Reh 2024) and by Torres et al. (2013) established core principles of gait sonification, demonstrating that acoustic transformation of kinematic variables can systematically alter gait symmetry, timing, and coordination in both healthy individuals and patients after unilateral hip arthroplasty. These approaches have implications for the design of interactive sonification displays and tangible auditory interfaces aimed at altering perceived and subsequent motor behaviour. Above, we already presented an example of early sensitivity to action-sound contingencies in infants through the sonification of spontaneous limb movements (Fig. 4B). Below, we consider several prominent examples in the context of movement, posture and locomotor control, illustrating how sonification can be used as an experimental tool to probe the principles of multisensory integration across development and adulthood.

A first line of work has examined how sonifying kinematic and dynamic parameters of movement can shape the acquisition of novel motor skills. In complex whole‑body actions such as rowing, providing real‑time auditory mappings of force production and segmental coordination enhances learning beyond rhythmic adjustments alone, leading to more accurate and stable movement patterns compared to visual or natural‑sound feedback (Effenberg et al. 2016). Similar benefits emerge in fine‑grained gesture learning: sonification reduces motor variability and accelerates the stabilization of complex trajectories, with stronger effects in individuals with musical training, suggesting that pre‑existing auditory expertise modulates the integration of movement‑related sound (Liu et al. 2022). Complementing these findings, recent work shows that different types of movement sonification shape not only kinematic execution but also the subjective experience of acting. In a mixed quantitative-qualitative study, sonifying upper‑limb movements systematically slowed and structured the temporal profile of the gesture while eliciting distinct experiential states, such as playfulness, imagery, and enhanced intentionality, depending on the sound mapping used (Peyre et al. 2023). This indicates that sonification acts not merely as an external cue but as an additional sensory channel that contributes to the formation and refinement of internal models of action.

A second line of evidence highlights the perceptual dimension of this process. Experiments manipulating the congruence and informativeness of movement‑related sounds show that sonification enhances the discrimination and recognition of gestures, and that this perceptual sharpening reciprocally improves motor execution (Bevilacqua et al. 2016). Conversely, performing actions with informative auditory feedback enhances the perceptual representation of those same gestures. This bidirectional relationship underscores the inherently multisensory nature of action control: perceiving movement more precisely supports better execution, and executing with enriched sensory consequences strengthens perceptual encoding (Effenberg 2005). As noted by Effenberg, these effects can be interpreted within a multisensory framework in which redundant auditory and proprioceptive cues enhance the precision of perceptual estimates. However, in the context of sonification this enhancement is more consistent with multisensory facilitation than with strict perceptual integration: movement‑related sounds typically function as biofeedback signals rather than as cues that are spatially bound to the moving limb. Consistent with this view, Boyer et al. (2013) showed that introducing a spatial conflict between the heard and actual hand position (a leftward shift of 18.5°) did not systematically modify pointing accuracy, suggesting that the CNS does not treat the different sensory information as originating from the same source. This interpretation remains compatible with physiological evidence showing that convergent multisensory inputs can amplify neural processing in heteromodal cortical regions, thereby improving perceptual reliability (Calvert et al. 2000).

**Fig. 5:**
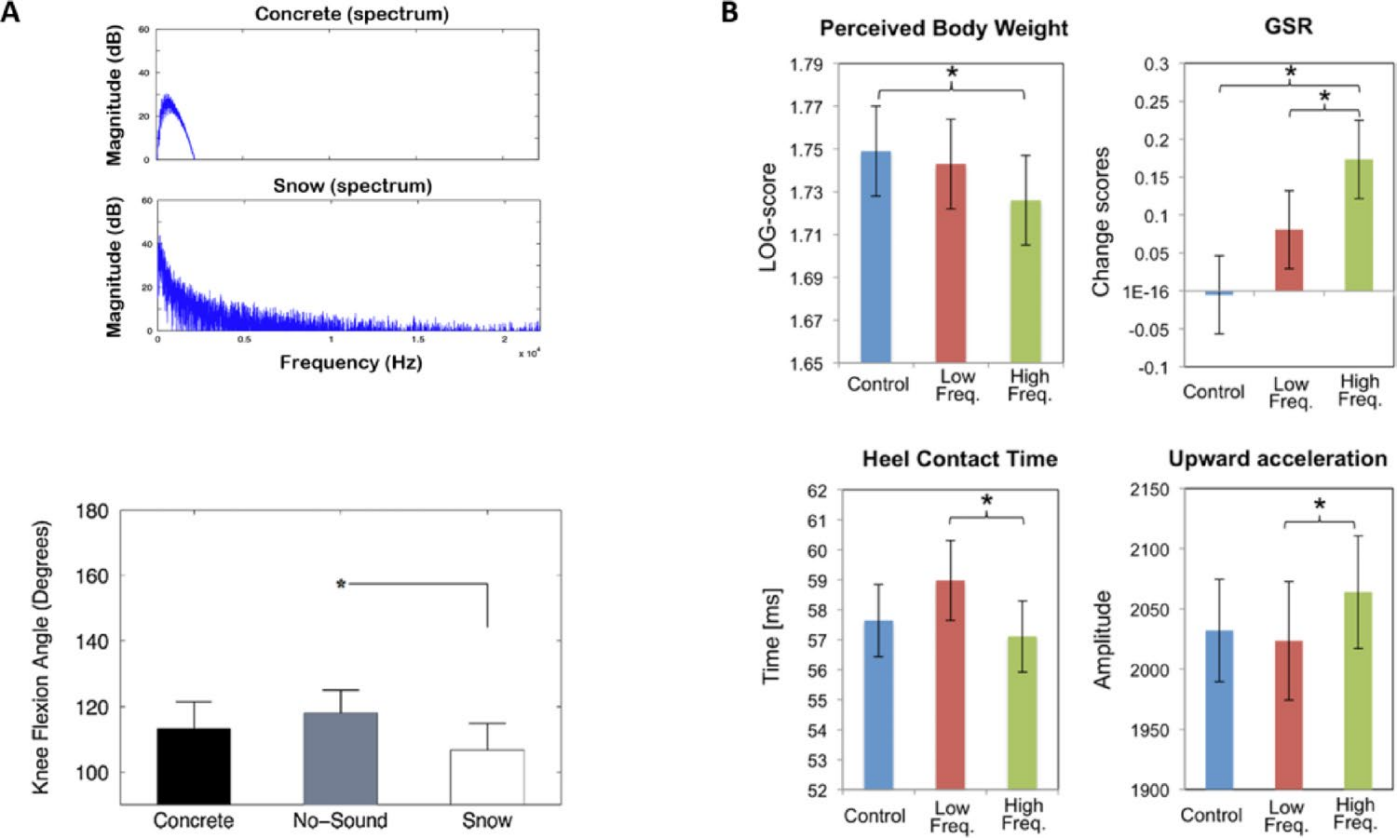
Examples of how manipulating footstep‑related sounds modulates locomotor perception and biomechanics. **A** From Turchet et al. (2015): typical frequency spectra of the two simulated walking surfaces (concrete and snow), and mean knee‑flexion angle across sound conditions (*p* < 0.05), showing reduced flexion when walking with “snow” sounds. **B** From Tajadura-Jiménez et al. (2015): mean (± SE) effects of Low‑, High‑frequency and Control footstep sounds on perceived body weight, galvanic skin response (GSR), heel‑contact time, and upward foot acceleration (marks significant differences). Low‑frequency sounds increased perceived body weight, heel‑contact duration, and physiological arousal, while high‑frequency sounds elicited greater upward acceleration and more positive emotional responses. Note that for GSR and questionnaire‑based affective ratings, lower scores reflect more negative emotional states, so the direction of the effect is inverted relative to the other measures

These principles extend to whole‑body actions such as walking. Manipulating footstep sounds in real time alters the perceived properties of the walking surface and induces measurable changes in gait dynamics, including aftereffects that persist once the manipulation is removed (Fig. 5A), evidence that auditory feedback is incorporated into the internal estimation of locomotor state (Turchet et al. 2015). Beyond surface perception, altering the spectral content of footstep sounds can shift perceived body weight, modulate emotional state, and reshape gait biomechanics, demonstrating that auditory feedback contributes to the multisensory construction of body representation and its coupling to affect and action. In the study of Tajadura-Jiménez et al. (2015), participants walked while hearing real‑time manipulations of their own footstep sounds, which were shifted toward higher or lower frequencies to evoke sensations of lightness or heaviness (Fig. 5B). These acoustic manipulations systematically altered perceived body weight, galvanic skin response, and gait parameters such as heel‑contact time and upward acceleration, indicating that footstep sounds are integrated into both the perceptual and motor estimation of the body in motion. A similar principle emerges in clinical populations: recent work shows that modifying the auditory consequences of footstep sounds can reshape both gait dynamics and the subjective experience of walking. In a multi-case study of individuals with chronic stroke, Matamala-Gomez et al. (2025) demonstrated that sonification of footsteps – using ‘wind-like’ or ‘mechanical’ sound textures – modulated perceived bodily lightness and flexibility while producing measurable changes in gait symmetry and walking speed. These findings reveal that the auditory consequences of self‑motion are deeply integrated into both the perceptual and motor dimensions of locomotion.

Consistent with this view, studies in the field of music performance analysis show that altering the reafferent sounds generated by one’s own actions can disrupt or reshape motor execution. For example, experimentally manipulated auditory feedback during piano performance induces systematic changes in timing and serial sequencing errors (Pfordresher 2005). Such effects have been interpreted within frameworks proposing shared predictive models for action and perception, whereby mismatches between expected and perceived auditory consequences dynamically influence subsequent motor output (Maes et al. 2014). This parallel suggests that, much like in musical performance, the auditory consequences of locomotor actions contribute to the ongoing calibration of movement through tightly coupled perception–action loops.

Finally, the same principles have motivated applications in rehabilitation. These experimentally induced auditory-motor couplings are not only informative about basic mechanisms, but also directly relevant for clinical contexts, where internal timing and multisensory integration are often compromised.

## Auditory-motor coupling in neurological and sensory disorders

Sonification of self-movement offers a means to provide external, behaviourally meaningful auditory scaffolds that can partially compensate for dysfunctional neural circuits. Recent user-centred studies emphasize that the clinical efficacy of gait sonification critically depends on the ecological relevance, intuitiveness, and perceptual salience of the auditory mappings. Kantan et al. (2022, 2023) showed that auditory feedback designed in close collaboration with clinicians and patients improves usability, movement awareness, and engagement during hemiparetic gait training, highlighting the importance of balancing informational richness with cognitive load.

Consistent with these findings, sonification systems that map gait parameters to intuitive auditory cues can enhance awareness of step symmetry, support corrective adjustments, and improve temporal stability during walking, offering a promising tool for restoring locomotor function in clinical populations. In line with this approach, Torres et al. (2013) propose a wearable system that transforms spatiotemporal gait parameters into real‑time auditory feedback, enabling users to detect asymmetries, adjust step timing, and stabilize their walking pattern. Similarly, Gomez-Andres et al. (2020) demonstrated that real‑time manipulation of footstep sounds in chronic stroke patients can modulate body‑perception cues, enhance movement awareness, and reduce gait asymmetries, indicating that auditory feedback can directly shape sensorimotor recalibration during walking.

Beyond locomotion, recent work has extended sonification-based feedback to upper‑limb rehabilitation: in a study comparing different sonification strategies in healthy and hemiparetic participants, continuous real‑time mappings of movement kinematics improved trajectory control, reduced variability, and were perceived as more intuitive and supportive than discrete or error‑based feedback (Peyre et al. 2023). By providing a manipulable and interpretable sensory channel, sonification enables targeted modulation of action-effect contingencies and offers a controlled framework for studying and shaping multisensory contributions to motor recovery.

Neurological disorders that affect the basal ganglia, cerebellum, or fronto‑striatal networks often compromise the internal generation of temporal predictions, resulting in unstable gait timing, increased variability, and impaired anticipatory control. Consistent with the broader framework outlined in recent reviews (Ghai and Ghai 2019; Braun Janzen et al. 2022; Scataglini et al. 2025), experimental and neuroimaging studies show that rhythmic auditory input can directly engage neural structures involved in temporal encoding and movement planning. Listening to a regular beat activates the premotor cortex, supplementary motor area, basal ganglia, and cerebellum, placing the motor system in a state of anticipatory readiness and enhancing the ability to predict the timing of upcoming events. This preparatory effect provides a temporally precise scaffold that supports the execution of cyclic movements such as gait. Beyond neurological disorders, sonification-based gait training has shown benefits in orthopaedic rehabilitation. Pietschmann et al. (2019) reported that real-time acoustic transformation of gait parameters after joint replacement promoted faster normalization of spatiotemporal gait patterns, suggesting that auditory feedback can accelerate recovery even when central timing mechanisms are preserved.

A key mechanism underlying this facilitation is the entrainment of neural oscillations, particularly within the beta frequency range (10–30 Hz), which plays a central role in motor timing, prediction, and the coordination of repetitive movements (Saleh et al. 2010). Beta‑band activity synchronizes with external rhythmic stimuli, linking auditory and motor cortices through fast, temporally precise coupling. In neurological disorders, where beta dynamics are often abnormal - excessively synchronized in Parkinson’s Disease (PD) or disorganized after stroke - rhythmic cues can restore more physiological oscillatory patterns. Clinical neurophysiological studies provide compelling evidence for this effect: rhythmic auditory stimulation during gait training increases beta‑band power and coherence across fronto‑central and temporal regions in PD (Calabrò et al. 2019), modulates beta oscillations within the subthalamic nucleus in patients with deep brain stimulation (Fischer et al. 2018), and enhances sensorimotor beta connectivity following rhythmic‑based motor training (Naro et al. 2020). These findings indicate that rhythmic stimulation enhances the entrainment, coherence, and phase‑specific modulation of beta‑band activity, restoring more physiological temporal dynamics essential for temporal prediction and movement stability, even when endogenous timing circuits are compromised. In Parkinson’s disease, real-time gait sonification has also been shown to induce short-term improvements in stride length, cadence, and gait regularity. In a pilot study, Gorgas et al. (2017) demonstrated that mapping gait parameters to continuous auditory feedback improved locomotor performance in people with PD, supporting the idea that sonification can complement rhythmic cueing by providing movement-specific information.

This neurophysiological evidence aligns with behavioural findings showing that rhythmic cueing can reconstruct impaired internal timing mechanisms. By providing a stable external beat, rhythmic stimulation enhances phase correction processes, strengthens temporal templates, and reduces gait variability, ultimately improving step regularity and inter‑limb symmetry. In the RESCUE trial, Nieuwboer et al. (2007) demonstrated that rhythmic cueing improved gait speed, step length, and freezing severity in PD despite basal ganglia dysfunction, with some benefits persisting even after cue removal. Manipulating the harmonic structure of auditory stimuli may modulate spatial gait parameters such as stride length and walking velocity in patients with Parkinson’s disease (Fritz et al. 2021). These results suggest that rhythmic input can bypass impaired fronto‑striato‑thalamo‑cortical loops and recruit alternative pathways to support locomotor timing. A complementary mechanistic account is provided by Nombela et al. (2013), who showed in their review that externally paced movements in PD rely on neural routes distinct from those used for self‑initiated actions. The authors argue that rhythmic cues can circumvent the dysfunctional basal ganglia-SMA circuit by engaging cerebello‑thalamo‑cortical pathways and auditory-premotor projections, effectively replacing the impaired internal clock with an externally driven temporal reference. This bypass mechanism explains why patients with severe deficits in internally generated timing can still synchronize their gait to rhythmic stimuli and why externally guided movements remain relatively preserved compared to self‑initiated ones.

Recent advances in wearable technology further extend these principles toward personalized rehabilitation. Within this growing line of work on individualized auditory feedback, Pang et al. (2024) developed a wearable, individualized sonification and biofeedback device that enhanced movement awareness and self-correction during daily activities, illustrating the potential of adaptive auditory feedback beyond laboratory settings. Complementary evidence shows that personalization can be achieved through different acoustic dimensions: cadence-dependent sonification based on real-time musical time-stretching improves step regulation more effectively than verbal instructions (Lorenzoni et al. 2018); pitch-based manipulations modulate bodily feelings, emotional valence, motivation, and movement kinematics (Ley-Flores et al. 2022); timbre-related manipulations, such as changes in harmonic complexity, modulate gait kinematics in neurological populations (Kim et al. 2020). Together, these findings illustrate that adaptive sonification can be individualized not only to motor deficits but also to perceptual preferences, supporting the development of ecologically relevant and user-centered rehabilitation tools.

Beyond these neurophysiological and behavioural findings, recent work has shown that auditory-motor coupling itself is not a static phenomenon but a dynamic process that fluctuates over time. Using a window‑based classification approach, Fayyad et al. (2024) demonstrated that individuals alternate between periods of stable step‑to‑beat synchrony (“locking”) and periods of desynchronization (“unlocking”) during continuous walking. These fluctuations were more pronounced in neurologically impaired participants, who exhibited longer and more frequent unlocking phases. Importantly, unlocking was associated with increased inter-step interval variability, indicating that instability in auditory-motor coupling directly translates into instability in gait timing. This fine-grained characterization suggests that neurological disorders not only impair internal timing mechanisms but also reduce the ability to maintain stable entrainment over time, making the coupling more fragile and susceptible to breakdown. Auditory-based interventions leverage the strong coupling between sound and movement to provide external spatial and temporal scaffolds that can stabilize posture and gait when internal multisensory and timing mechanisms are compromised.

## Conclusions

The evidence reviewed here demonstrates that auditory information is a core component of postural and locomotor control, rather than a secondary or contextual cue. Sound provides spatial, temporal, and predictive information that is continuously integrated with vestibular, visual, and somatosensory inputs to stabilize the body and organize movement. This integration operates across multiple levels of the nervous system, from the shared mechanosensory architecture of the inner ear and brainstem to distributed cortical and subcortical networks that jointly encode sound, self‑motion, and motor state. A key theme is the tight functional coupling between the auditory and vestibular systems. Their common developmental origin, overlapping peripheral and central pathways, and shared efferent control mechanisms support cross‑modal calibration of spatial orientation and movement. Auditory stimuli can influence vestibular processing directly, while vestibular and locomotor signals dynamically shape auditory perception, supporting active auditory sensing during movement. Together, these interactions provide a flexible multisensory reference frame for balance, navigation, and gait. Importantly, these effects depend on the spectral, temporal, and spatial properties of sound, indicating selective exploitation of informative acoustic features.

The translational relevance of these mechanisms is evident in neurological and sensory disorders characterized by impaired internal timing or multisensory integration. Rhythmic auditory stimulation and movement sonification can provide external spatial or temporal scaffolds that partially compensate for dysfunctional neural circuits, engaging oscillatory and network‑level mechanisms that support prediction and coordination. Overall, recognizing audition as an integral contributor to posture and locomotion refines our understanding of multisensory motor control and opens new perspectives for auditory‑based interventions. Future work combining developmental, mechanistic, and ecologically valid approaches will be essential to optimize auditory strategies for balance training, gait rehabilitation, and fall prevention.

Looking forward, several challenges and opportunities emerge. A more systematic characterization of how specific acoustic parameters interact with vestibular and somatosensory processing is needed, particularly in ecologically valid settings. Longitudinal and developmental studies could clarify how early auditory‑motor couplings contribute to the multisensory calibration processes that support the later development of balance and gait abilities. Finally, advances in wearable technology and adaptive sonification open new possibilities for personalized, multisensory rehabilitation strategies that leverage the inherent coupling between sound and movement.

## Data Availability

No datasets were generated or analysed during the current study.
